# Automatic analysis of 2D polyacrylamide gels in the diagnosis of DNA polymorphisms

**DOI:** 10.1186/1475-925X-12-68

**Published:** 2013-07-08

**Authors:** Robert Koprowski, Zygmunt Wróbel, Anna Korzyńska, Karolina Chwiałkowska, Mirosław Kwaśniewski

**Affiliations:** 1 Department of Biomedical Computer Systems, Institute of Computer Science, University of Silesia, Będzińska 39, 41-200, Sosnowiec, Poland; 2 Nałęcz Institute of Biocybernetics and Biomedical Engineering, Polish Academy of Sciences, Ks. Trojdena 4, 02-109, Warszawa, Poland; 3 Department of Genetics, University of Silesia, Jagiellońska 28, 40-032, Katowice, Poland

**Keywords:** Gel, Image processing, Polyacrylamide, Segmentation, 2D

## Abstract

**Introduction:**

The analysis of polyacrylamide gels is currently carried out manually or automatically. In the automatic method, there are limitations related to the acceptable degree of distortion of lane and band continuity. The available software cannot deal satisfactorily with this type of situations. Therefore, the paper presents an original image analysis method devoid of the aforementioned drawbacks.

**Material:**

This paper examines polyacrylamide gel images from Li-Cor DNA Sequencer 4300S resulting from the use of the electrophoretic separation of DNA fragments. The acquired images have a resolution dependent on the length of the analysed DNA fragments and typically it is *M*_*G*_×*N*_*G*_=3806×1027 pixels. The images are saved in TIFF format with a grayscale resolution of 16 bits/pixel. The presented image analysis method was performed on gel images resulting from the analysis of DNA methylome profiling in plants exposed to drought stress, carried out with the MSAP (Methylation Sensitive Amplification Polymorphism) technique.

**Results:**

The results of DNA polymorphism analysis were obtained in less than one second for the Intel Core™ 2 Quad CPU Q9300@2.5GHz, 8GB RAM. In comparison with other known methods, specificity was 0.95, sensitivity = 0.94 and *AUC* (Area Under Curve) = 0.98.

**Conclusions:**

It is possible to carry out this method of DNA polymorphism analysis on distorted images of polyacrylamide gels. The method is fully automatic and does not require any operator intervention. Compared with other methods, it produces the best results and the resulting image is easy to interpret. The presented method of measurement is used in the practical analysis of polyacrylamide gels in the Department of Genetics at the University of Silesia in Katowice, Poland.

## Introduction

**Figure 1:**
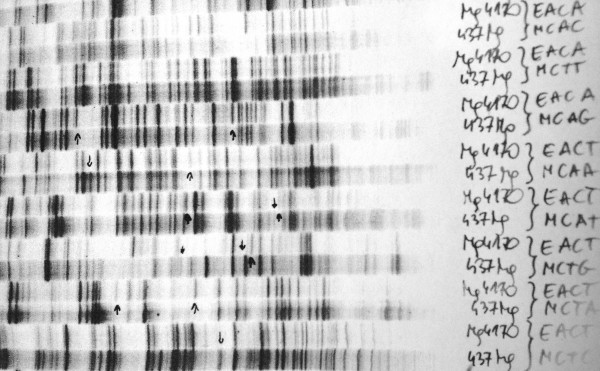
**Manual analysis of polyacrylamide gels in the distorted polyacrylamide gel image.** The operator manually selects interesting bands. Their location, number, and correlation with other bands detected manually are recorded in the margin. Because of characteristic distortion, common software cannot deal with the arrangement of individual lanes in these images.

Modern DNA analysis is used in many areas of life sciences, from biology [1] to forensic medicine or microwave analysis [2,3]. For many such cases, the analysis of DNA is associated with electrophoresis carried out on polyacrylamide gels, an universal analytical technique used to separate DNA fragments by size. The advantages of using polyacrylamide gels are low cost of staining separated DNA fractions and also easily interpretable analysis results. The obtained results are compared manually or semi-automatically. The manual method involves a manual selection of lanes and bands that are in the analysed area. Most often this occurs by selecting interesting bands on the printed analysis result (Figure 1) or by placing the lines along the lane on the computer screen. This analysis involves finding the location of the subsequent bands along the lane that was marked by the operator. In this case, errors occur:

•due to different print quality depending on the type of printer (contrast, type of paper used etc.),

•related to the participation of human factors, i.e.: the impact of experience, fatigue, sensitivity to illusions related to the impact of the expected result on the course of the analysis and an individual threshold of distinguishing bands from the background.

A major difficulty is also the amount of time devoted to the same analysis and the lack of reproducibility of measurements.

Known methods and software for automatic analysis have disadvantages mainly related to the analysis of gel images in which individual bands are not located on a straight lane (Figure 1). There are also problems with the proper separation of lanes and detection of bands which are arranged close to each other. The analysis of the DNA fragments, observed as bands on a gel image, can thus be divided into two parts: the separation of lanes and the separation of bands in each lane. The result of detection of band positions is most often the matrix *L*_*DNA*_ containing the value "1" in the places where a band occurs and "0" in the other places. The number of rows of the matrix *L*_*DNA*_ corresponds to the number of positions of all the bands, and the number of columns corresponds to the number of gel lanes [4,5]. Since the matrix *L*_*DNA*_ is, by definition, a binary matrix, further analysis and comparison of results for subsequent lanes is easy. Therefore, a key issue is appropriate separation of lanes and bands for each lane related to image analysis and processing.

The first works on the analysis and processing of polyacrylamide gel images obtained from electrophoresis are from the 80's, for example, the works of L. Lipkln [6] or Stanley et al. [7]. These relate to the basic methods of analysis of image brightness for each lane. The authors of [8] does not include any information on how to separate individual lanes. The authors assume that they are arranged perfectly parallel. Similar assumptions are in [9,10]. The authors of [11] from 2001 present the analysis of individual bands using information about the brightness gradient. Bands are defined depending on the distance between the changes of the gradient sign. This method is useless when two neighbouring bands are connected or there is uneven brightness on the whole gel. In other works, different methods of image analysis and processing are used, e.g.: active contour [12], the Gaussian distribution [11], fuzzy c-means algorithm [13] or statistical analysis [14]. Another group of works is devoted to the development of these methods. For example, the works of J. Pizzonia [15] and L. Carol [16], GILE software (Gel-Image-Extractor) [17] or [18-23]. In [18], gels in large scale were analysed, [19] used the method of least squares, and [20] shows a method of using morphological operations (erosion) in the analysis of ROI (Region Of Interest) of gels. The aforementioned GILE software [17] is not the only available software. There are other applications for automatic or semi-automatic analyses of 2D gels, such as GelQuant [24], GelAnalyzer [25], Gel-Pro Analyzer [26], Decodon [27], BioNumerics 2D [28], Delta2D [29], ImageMaster 2D, Melanie [30], PDQuest [31], Progenesis Samespots [32] or REDFIN [33] and many others. A wide range of available programs for gel image analysis enables to obtain satisfactory results in the case of simple gels with individual lanes arranged in parallel. If there are artefacts, connected lanes or bands, this group of software [19,24-33] allows for their manual editing. In these cases, the method is semi-automatic or fully manual. Therefore, more sophisticated methods of image analysis must be used or the analysis algorithm must be profiled precisely to the specified problem (a given type of gels). One such method proposed by the authors is described below. It is characterized by a new approach to the analysis of polyacrylamide gels which provides: fully automatic measurement of the band position, automatic determination of the lane position in cases of their local distortion, results in the form of a matrix of band occurrences (for all lanes). A special feature that distinguishes the approach presented below from other well-known methods, is the correct algorithm operation in cases of changes in lane thickness.

## Material

This paper examines polyacrylamide gel images from Li-Cor DNA Sequencer 4300S resulting from the use of the electrophoretic separation of DNA fragments. The acquired images have a resolution dependent on the length of the analysed DNA fragments and typically it is *M*_*G*_×*N*_*G*_=3806×1027 pixels. The images are saved in TIFF format with a grayscale resolution of 16 bits/pixel. The images of banding patterns of DNA amplification products were obtained after digestion with two enzymes, namely *Hpa*II and *Msp*I, used in the DNA methylome profiling method, MSAP (Methylation Sensitive Amplification Polymorphism).

The analysis was performed on DNA isolated from plants exposed to drought stress at four time points *t*_*1*_, *t*_*2*_, *t*_*3*_ and *t*_*4*_ and from control plants at one time point (*t*_*5*_), which gave a total of 5 points (the first two time points *t*_*1*_ and *t*_*2*_ are shown in Table 1). At each time point, DNA of 3 plants was analysed (*biol*_*1*_*, biol*_*2*_ and *biol*_*3*_ – Table 1). The reaction was carried out in two technical replicates for each enzyme, for a total of four trials for each of the three biological replicates. The trials are arranged on gels vertically in successive lanes according to Table 1. Banding patterns were analysed by assessing the presence or absence of a band for a given track by transforming the gel image into a matrix consisting of "0" or "1", where "0" means no band and "1" means its presence. Further analysis involves designation of the dominant banding pattern for all replicates, both the technical as well as biological ones. What is subject to assessment are the differences in banding patterns between the points *t*_*1,*_*t*_*2,*_*t*_*3,*_*t*_*4*_ and *t*_*5*_. A total of 20 gel images were analysed in each of 60 tracks with different levels of distortion.

**Table 1:** The arrangement of biological and technical replicates on the gel - the first two time points

**Time point**	***t***_***1***_												***t***_***2***_											
**biological repeat**	***biol***_***1***_				***biol***_***2***_				***biol***_***3***_				***biol***_***1***_				***biol***_***2***_				***biol***_***3***_			
**Enzyme**	***Hpa*II**		***Msp*I**		***Hpa*II**		***Msp*I**		***Hpa*II**		***Msp*I**		***Hpa*II**		***Msp*I**		***Hpa*II**		***Msp*I**		***Hpa*II**		***Msp*I**	
**technical repeat**	1	2	1	2	1	2	1	2	1	2	1	2	1	2	1	2	1	2	1	2	1	2	1	2
**trial number**	1	2	3	4	5	6	7	8	9	10	11	12	13	14	15	16	17	18	19	20	21	22	23	24

## Implementation of the new method

The new method, proposed by the authors, is based on the analysis of gel images which is carried out in two separate stages: analysis of lanes and analysis of bands. In both stages there are similar problems with the detection of objects and the removal of their redundancy (interference). Both the first analysis and the latter one require pre-filtration of the image and normalization described below.

### Preprocessing

In the first stage, the image *L*_*G*_ with a resolution of *M*_*G*_×*N*_*G*_=3806×1027 pixels is subjected to filtration with a median filter whose mask size is 3×3 pixels (the result is the image *L*_*F*_) [34-36]. The median filter mask size was chosen based on the image resolution and the size of possible artefacts present in the image (minor defects, CCD errors and noise of the electronics). In a further step, the image *L*_*F*_ undergoes normalization operation from the range of brightness levels corresponding to 16 bits (2^16^) to the floating range 0–1. The image *L*_*O*_ thus obtained is subjected to further processing steps.

### Processing – the analysis of lanes

The input image *L*_*O*_, after filtration and normalization, was subjected to the analysis of lanes. It involves the operation of closure with a structural element *SE* sized *M*_*SE*_×*N*_*SE*_=40×1 pixels [37-41]. The size of the structural element *SE* was chosen in such a way as to highlight the changes in brightness between individual lanes in the gel image. When decreasing the resolution *M*_*SE*_, the contrast between individual lanes decreases. An increase in the resolution *M*_*SE*_ causes the loss of information about the angle changes of the lane. When increasing *N*_*SE*_, on the other hand, the contrast between adjacent lanes decreases.

**Figure 2:**
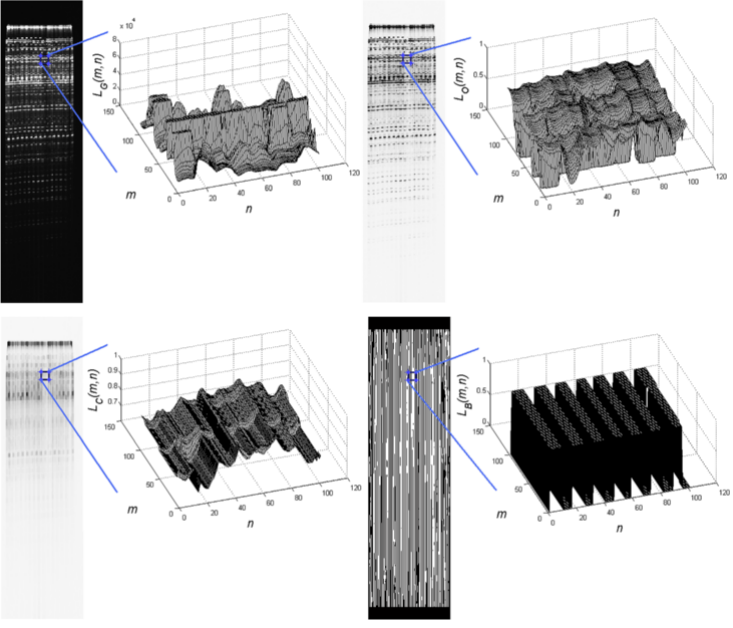
**Gel input image and subsequent stages of the lane analysis.** From the left: the input image *L*_*G*_, the image *L*_*O*_ after filtration and normalization, and below, the image *L*_*C*_ after opening and the image *L*_*B*_ which is a binary image with automatically marked lanes. Due to the high resolution of the presented images, on their right side there is a chart that shows changes in brightness of a fragment.

**Figure 3:**
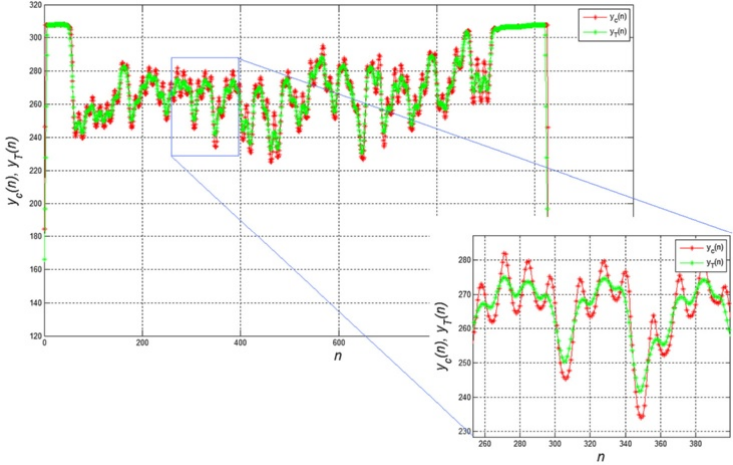
**Examples of functions of changes in brightness for the row of the image matrix *L***_***C***_**.** There are shown differences between *y*_*C*_(*n*) and *y*_*T*_(*n*) which provide valuable information about local changes in brightness. Depending on the value of these differences, decisions are made about the detection of lanes whose width must fall within the adopted range. Narrower objects are considered as interference and the wider are split into smaller ones.

The resulting image *L*_*C*_ is further analysed in the subsequent rows. For each row in the range *m*∈[0,*M*_*G*_], level differences of brightness *y*_*C*_(*n*) are analysed in relation to the filtered function *y*_*T*_(*n*) of *y*_*C*_(*n*). The value of *y*_*C*_(*n*) is brightness for the selected *m* i.e. *y*_*C*_(*n*) =*L*_*C*_(*m,n*) for *m*=const, while the value of *y*_*T*_(*n*) results from the filtration of *y*_*C*_(*n*) with an averaging filter sized 1×10. The averaging filter size is chosen once and is equal to the typical lane width, which in this case is 10 pixels. The resulting differences are shown in Figures 2 and 3 where differences below zero (black bands in the white background) form the white pixels and the other ones form black pixels. The resulting image *L*_*B*_ is the final stage of the analysis of lanes. For further analysis, it will be used in conjunction with the source image *L*_*O*_.

### Processing – the analysis of bands

**Figure 4:**
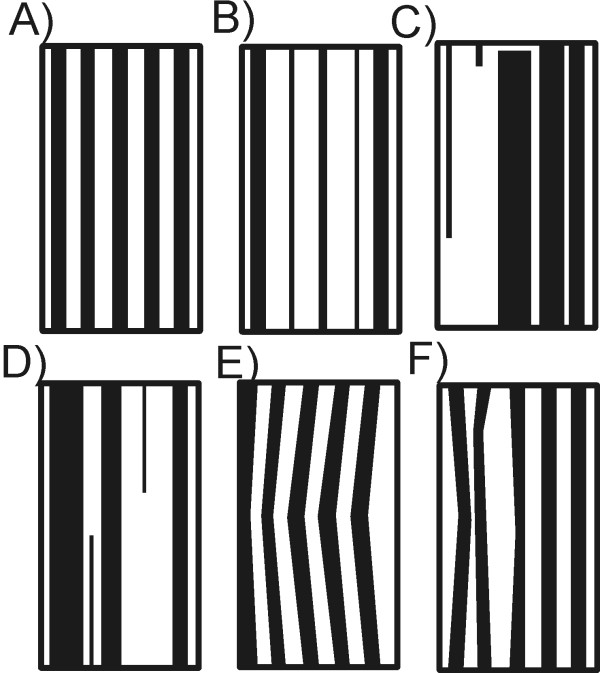
**Image showing various artefacts that occur when analysing lanes.** The images show the following cases: **A**) – correct, the lanes are continuous and have a fixed width, **B)** – incorrect, the lanes have uneven width, **C**), **D**) - incorrect, the lanes are broken and their proper identification and classification, without further analysis, is difficult, **E**) the lanes are not arranged in a straight line, **F**) the lanes do not have equal width.

**Figure 5:**
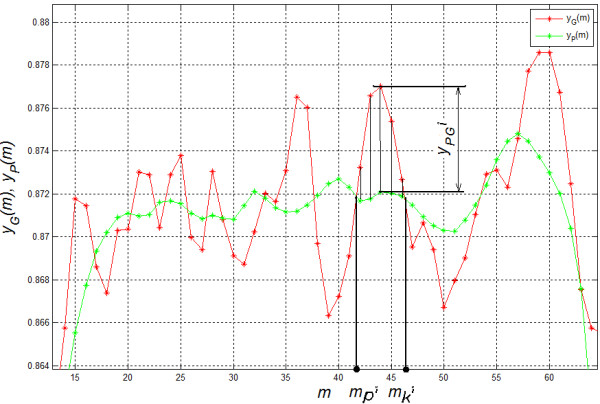
**Changes in the brightness of a selected lane in the image *L***_***O***_**.** The difference between the brightness *y*_*P*_(*m*) calculated along the lane and its averaged result *y*_*G*_(*m*) is vital. Depending on the result of *y*_*P*_(*m*)-*y*_*G*_(*m*), the position of markers indicating subsequent bands is determined. The chart shows the range *m*_*k*_-*m*_*p*_ which is the width of a sample band.

The analysis of band positions requires detection of each lane individually. This means that it should be possible to separate each lane in the image *L*_*B*_. Unfortunately, in practice, due to the existence of artefacts, a complete separation of each lane is impossible. The lanes visible in the image *L*_*B*_ are often linked together, interrupted or terminated in the wrong place - Figure 4[42-46]. Due to such situations, the analysis of individual bands and their location for individual lanes must be performed in smaller ROIs. The ROI size must be no less than the width of a single lane and not greater than an average distance between the artefacts. In practice, it appears that the best results are obtained for ROI sizes that are 10 to 20 times the width of the lane [47,48]. In the analysed case, it is *M*_*ROI*_×*N*_*ROI*_=200×200 pixels. The image *L*_*B*_ is divided into *M*_*G*_*/M*_*ROI*_ in rows and *N*_*G*_*/N*_*ROI*_ in columns*.* In total, for the resolution of the image *L*_*G*_ equal to *M*_*G*_×*N*_*G*_=3806×1027 pixels, there are, after rounding, 95 ROIs for the analysis. For each ROI, the lanes were labelled, which gave the image *L*_*IND*_. Then, band detection is carried out for each lane in an analogous manner to lane detection [49-51]. The difference between the brightness *y*_*P*_(*m*), calculated along the lane, and its averaged result *y*_*G*_(*m*) are analysed. The averaging area of the input waveform *y*_*P*_(*m*) is chosen once, individually for each device. This area is dependent on the *L*_*G*_ image resolution and typical (average) lane width. For the case described, it is the area of 10 pixels. Depending on the result of *y*_*PG*_(*m*)=*y*_*P*_(*m*)-*y*_*G*_(*m*), the location of markers which indicate the subsequent bands is determined. Two features are considered here: the maximum difference *y*_*PG*_^*i*^*=max*(*y*_*PG*_(*m*)) for *m∈*[*m*_*p*_^*i*^*,m*_*k*_^*i*^] where *m*_*p*_^*i*^*,m*_*k*_^*i*^ are the beginning and end of the *i*-th band and the width of the band Δ*m*^*i*^*= m*_*k*_^*i*^- *m*_*p*_^*i*^. The band feature based on the pair *y*_*PG*_^*i*^ and Δ*m*^*i*^ can be compared to the area under the curve *y*_*PG*_^*i*^ - Figure 5. The parameter Δ*m*^*i*^, which enables the separation of combined bands, will be used in further analysis. In practice, it is most convenient to adopt the acceptable range of variation of Δ*m*^*i*^ covering the range of 50-150% of a typical band width. Below this range (<50%), a detected object is considered to be interference, whereas above this range (>150%) a detected object is considered as a combination of two bands. Another considered parameter *p*_*r*_ of thresholding *y*_*PG*_^*i*^ was chosen on the basis of the analysis of sensitivity *SPC* and specificity *TPR*. The values of sensitivity and specificity were determined by comparing the performed automatic analysis with the manual analysis carried out by an expert for 20 images containing 60 lanes each. Depending on the detection or omission of a band in any of the trials, the results were determined as false negative *FN*, false positive *FP*, true negative *TN* and true positive *TP*. The obtained results of *FN*, *FP*, *TN*, *TP* for the optimal value of *p*_*r*_=9% are shown in Table 2 (*SPC*=0.95, *TPR*=0.94).

**Table 2:** **Results of *FP, FN, TP, TN, SPC and TPR *obtained for the threshold *p***_***r***_** changed in the range from 0 to 30%**

***p***_***r***_**[%]**	***FP***	***FN***	***TP***	***TN***	***SPC***	***TPR***
0	61698	67	6316	1022	0.01	0.98
1	29680	76	6307	32921	0.52	0.98
2	19360	81	6302	43221	0.69	0.98
3	15197	81	6302	47378	0.75	0.98
4	11452	81	6302	51117	0.81	0.98
5	8746	88	6295	53821	0.86	0.98
6	6762	111	6272	55801	0.89	0.98
7	5006	111	6272	57550	0.91	0.98
8	3821	192	6191	58734	0.93	0.96
**9**	**2603**	**321**	**6062**	**59952**	**0.95**	**0.94**
10	1914	471	5912	60639	0.96	0.92
11	1144	615	5768	61409	0.98	0.90
12	759	690	5693	61792	0.98	0.89
13	502	766	5617	62049	0.99	0.87
14	303	887	5496	62248	0.99	0.86
15	0	1010	5373	62548	1	0.84
16	0	1193	5190	62548	1	0.81
17	0	1786	4597	62548	1	0.72
18	0	2206	4177	62548	1	0.65
19	0	2642	3741	62548	1	0.58
20	0	3126	3257	62548	1	0.51
21	0	3300	3083	62548	1	0.48
22	0	3968	2415	62548	1	0.37
23	0	4454	1929	62548	1	0.30
24	0	4806	1577	62548	1	0.24
25	0	5090	1293	62548	1	0.20
26	0	5220	1163	62548	1	0.18
27	0	5249	1134	62548	1	0.17
28	0	5307	1076	62548	1	0.16
29	0	5329	1054	62548	1	0.16
30	0	5382	1001	62548	1	0.15

**Figure 6:**
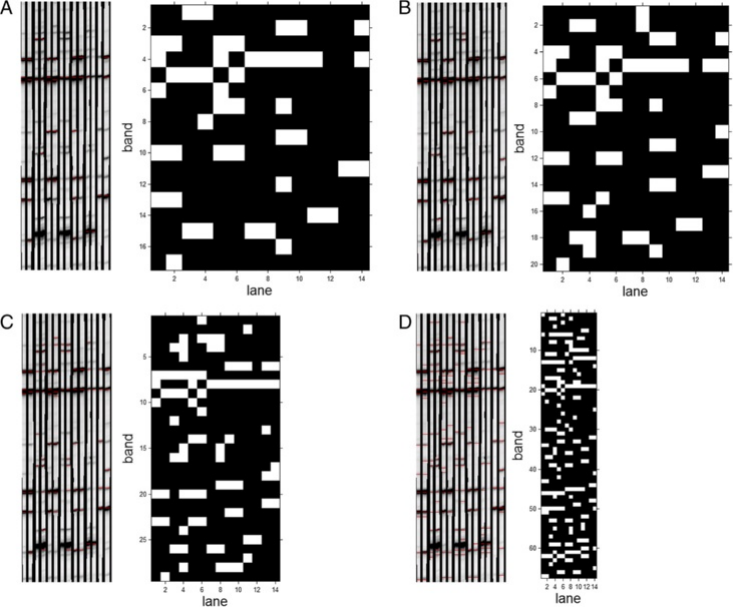
**Input image with marked and identified bands and the output matrix *L***_***DNA***_**for A) *p***_***r***_**=0.2, B) *p***_***r***_**=0.4, C) *p***_***r***_**=0.6, D) *p***_***r***_**=0.8.** Each row of the matrix *L*_*DNA*_ is the band position and the column is the subsequent lane. The matrix *L*_*DNA*_ can be directly compared for selected columns. The comparison concerns the assessment of conformity of performed technical and biological tests and the differences between the structure of the DNA. The white pixels indicate a recognized band and the black ones indicate the background.

The last stage of the analysis is the conversion of individual band positions to the matrix *L*_*DNA*_ in which columns correspond to subsequent lanes and rows to the location of subsequent bands - Figure 6. This matrix is formed as a result of the conversion of the band and lane coordinates (*m*_*s*_^*i*^*,n*_*s*_^*i*^) for the adopted *y*_*PG*_^*i*^ and Δ*m*^*i*^. This matrix is further verified in terms of reproducibility between technical replicates (Table 1), biological replicates and finally differences in the DNA structure. Then, these differences are easy to mark automatically (a comparison of adjacent columns with the operation xor). In this case, the matrix *L*_*DNA*_ has a constant number of columns equal to the number of lanes, whereas the number of rows is variable and depends on the threshold value, namely *p*_*r*_. This matrix has a resolution of 5×60 to 200×60 pixels for typical conditions.

The next section shows the comparison of the quality of the obtained results with other methods described in other works.

## Comparison with other methods

**Figure 7:**
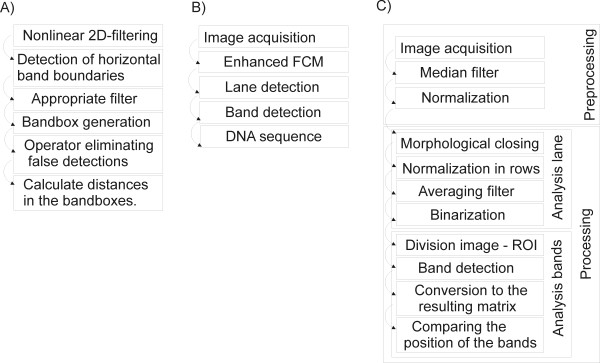
**Algorithm block diagrams. A**) - the algorithm proposed in this article, **B**), **C**) – other compared algorithms proposed by I. Bajla in [5] and Jiann-Der Lee in [11]. Visible differences relate primarily to the main analysis of data. In the proposed method, the analysis consists of two steps: lane detection followed by band detection. In the other compared methods, the main element of the algorithm is band detection, whereas lane identification is neither analyzed nor presented.

The comparison of the quality of the results was carried out on 20 available gel images containing 60 lanes each, which gave a total of 1200 lanes. Two image analysis algorithms, known from the literature, were implemented; method 1 - [5] and method 2 - [11]:

Method 1 – proposed by I. Bajla et al. in [5] – Figure 7A. This method involves filtration with a non-linear two-dimensional filter. Then, lane detection, smoothing and the analysis of peaks in the background area of the lane are carried out in the resulting image. In the next step, an operator manually corrects false results. Obtained results are shown in the form of bands in a gel diagram. This method is not fully automatic. The operator must manually correct the falsely detected bands. The number of wrongly identified bands varies and is highly dependent on the operator’s individual features, mainly contrast threshold below which the band is considered as interference.

Method 2 – proposed by Jiann-Der Lee et al. in [11] – Figure 7B. The analysis of images is as follows. After entering the image, there is lane detection followed by band detection. Band detection is carried out on the basis of the enhanced fuzzy c-means algorithm. The authors suggest a nonlinear estimator based on the Gaussian kernel transformation. The position of the band, which is treated as an ellipse of varying sizes, is recognized in the range proposed by the authors. The analysis errors obtained by the authors for the database of gel images described in [11] are 6.7% and 12.8% when using the N. Otsu thresholding method [52].

Method 3 – described in this paper – Figure 7C.

All 3 methods were implemented according to the descriptions in [5,11] and in accordance with the block diagrams shown in Figure 7A,B and C. The results depend on three elements: the degree of lane tortuosity, the threshold of band distinction and shifts in the position of bands for individual lanes.

The degree of lane tortuosity influences, to a significant extent, the distinction error of the band position in the axis *0x*. The consequence of winding lanes or their uneven thickness is that the error of the correct assignment of a band to a lane for the 1200 analysed lanes for methods 1 and 2 is very large and highly dependent on the amplitude of lane tortuosity (Figure 4E). In method 3, discussed in this paper, the error does not exceed a few per cent (the exact comparison is carried out in the next paragraph). The advantage of method 3 over methods 1 and 2 results from the lane area analysis. However, the problem with method 3 is an appropriate separation of lanes when the shift does not fall within the range of the ROI analysis. Although methods 1 and 2 enable manual correction of the obtained results, it consumes a lot of time and requires operator intervention in the results. Method 3, on the other hand, is fully automatic.

The threshold of band distinction is highly dependent on the adopted methodology of gel image analysis. In method 1, the band width on one-dimensional waveform is specified. The changes in the median values in front of and behind the band are analysed. Based on this comparison, a new band is recognized on a given lane. Due to the constant band width adopted in method 1 Δ*m*^*i*^=const. This method cannot deal with the proper detection of bands which are close to each other, especially in situations when the combined total width of the bands is not close to a multiple of their width (2*Δ*m*^*i*^), and these situations often occur in practice [50,51,53]. In method 2, two closely situated bands are well separated even when their combined width is not equal to a multiple of their width.

**Figure 8:**
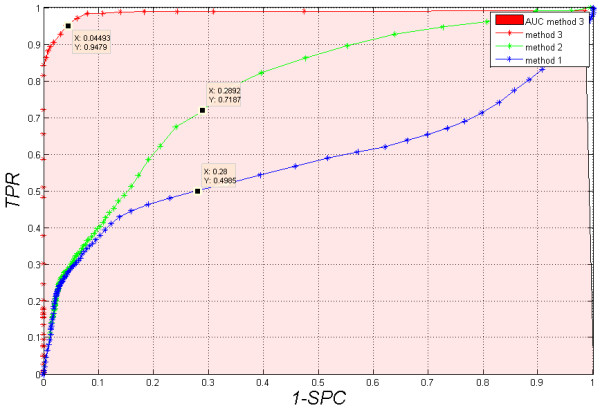
**ROC graph.** Dependency of sensitivity and specificity changes for the changes in the cut-off threshold *p*_*r*_ for methods 1, 2 and 3 without manual correction of the results. Changes in *p*_*r*_ are in the range from 0 to 1 for the compared methods 1, 2 and 3. The best results were obtained for method 3 presented in this article. Methods 1 and 2 have worse results. For manual correction of the results, possible in methods 1 and 2, the obtained results are perfect.

In each method, the selection (manual or automatic [52,54]) of the brightness threshold *p*_*r*_, that determines the visibility of bands, becomes dominant. As a result, for methods 1, 2 and 3, the threshold *p*_*r*_ was changed while observing changes in specificity and sensitivity of the ROC curves (Receiver Operating Characteristic). The results are shown in Figure 8. The best results were obtained for method 3, i.e.: *SPC*=0.95, *TPR*=0.94. In the case of methods 1 and 2, low sensitivity and specificity are due to the lack of manual correction of the results. For method 1, specificity and sensitivity are as follows: *SPC*=0.72, *TPR*=0.5. For method 2, they are: *SPC*=0.71, *TPR*=0.72. Manual correction of the results obtained in methods 1 and 2 improves the results to the ideal values. Therefore, in the case of manual correction of the results, they are always only slightly better than the results obtained from the presented automatic method 3 (*SPC*=0.95, *TPR*=0.94 and *AUC*=0.98) – Table 3.

**Table 3:** Comparison results of the three methods for the analysis of polyacrylamide gel images

**Method**	**Author**	***SPC***	***TPR***
1	Bajla et al. [5]	0.72	0.50
2	Jiann-Der Lee et al. [11]	0.71	0.72
3	Koprowski et al. this article	0.95	0.94

Comparing the results for the analysed lanes of all gels, the following conclusions can be drawn:

- method 1 enables to obtain satisfactory results for little complex analysis in which there is no need to analyse lane tortuosity; it is possible to manually correct the incorrect results,

- method 2 enables to obtain good results for complex analysis; it is possible to manually correct the incorrect results,

- method 3 enables to obtain good results fully automatically even in the case of winding lanes; it is fully automatic.

In addition to methods 1 and 2, the described method 3 can be compared with many other known methods. These are the ones mentioned in the introduction, or for example, the method described in detail by Caridade C. in [55]. The author describes the method (GEIAS) of gel image analysis and the correction of their wrong position - rotation. For 12 images (1082 bands in total) analysed in [55] the error is 9.2%. The images are also rotated in the angular range from -10° to 10° at 0.5° increments. The resistance of the proposed algorithm GEIAS to rotation is then analysed. The method described in [55] does not cover the width distortion of individual lanes which often occurs in practice. It is one of the major differences in comparison to method 3 proposed in this paper.

Method 3, in the described cases, enables to obtain the best results, but it does have its drawbacks. These include:

- limited resistance to lane width distortion - especially in situations when they occur together with the noise of image acquisition,

- limited resistance to the decay of individual lanes - for example due to the errors in image acquisition or other (biological) factors,

- the need for single introduction of selected parameters of the algorithm for each new gel image acquisition device.

Elimination of these defects is difficult in practice and will be the subject of the authors’ future works.

## Conclusions

The proposed methodology for the analysis and processing of polyacrylamide gel images enables to perform an automatic and repeatable measurement of the position of lanes and bands. This method is superior to the previously presented methods, described in [5] and [11], in cases of gel deformation. In such situations, each track width is different and it is difficult to identify to which lane the recognized band belongs.

Proper identification of bands and lanes in the described method enables to obtain the output image *L*_*DNA*_. This image (*L*_*DNA*_) contains information about the band positions in rows and information about the lane positions in columns. The number of recognized bands is highly dependent on the threshold *p*_*r*_ which is determined manually or automatically. The best results (*SPC*=0.95, *TPR*=0.94 and *AUC*=0.98) were obtained for *p*_*r*_=8%. The results are worse for other threshold values (Figure 6).

The presented measurement method is used in practical analysis of polyacrylamide gels in the Department of Genetics at the University of Silesia in Katowice, Poland.

Further studies concern the correction of the obtained results shown in Figure 6. In the case of differences between repeats shown in the image *L*_*DNA*_, it is necessary to move back to the image *L*_*O*_ and analyse the brightness of the area of interest. Depending on the comparison, correction must be made in the image *L*_*DNA*_. However, this method suggests a distinct approach to the analysis of lanes that involves the comparison of adjacent lanes directly in the image *L*_*O*_, which requires separate studies.

## Abbreviations

GILE: Gel-Image -Extractor; SPC: Specificity; TPR: sensitivity; TP: True positive; TN: True negative; FN: False negative; FP: False positive; ROC: Receiver Operating Characteristic; ROI: Region Of Interest.

## Competing interests

The authors declare that they have no competing interests.

## Authors’ contributions

RK suggested the algorithm for image analysis and processing, implemented it and analysed the images. ZW, AK, KC and MK performed the acquisition of the gel images and consulted the obtained results. All authors have read and approved the final manuscript.

## References

[B1] Korzyńska A Hoppe A Strojny W Investigation of a combined texture and contour method for segmentation of light microscopy cell images 2004 Biomedical Engineering: Proceedings of the Second IASTED International Conference on 234 239

[B2] Foster KR Comments on DNA as a fractal antenna Int J Radiat Biol 2011 87 12 1208 1209 10.3109/09553002.2011.626490 22132741

[B3] Foster KR Epstein BR Gealt MA “Resonances" in the dielectric absorption of DNA? Biophys J 1987 52 3 421 425 10.1016/S0006-3495(87)83231-9 3307930 PMC1330007

[B4] Patel D Gel electrophoresis: Essential data 1994 New York: Wiley

[B5] Bajla I Holländer I Burg K Improvement of Electrophoretic Gel Image Measurement Science Review 2000 1 1 5 10

[B6] Lipkln L Lemkin PF Data-Base Techniques for Multiple Two-Dimensional Polyacrylamide Gel Electrophoresis Analyses Clin Chem 1980 26 10 1403 6996868

[B7] Stanley KK Pitt TJ Quantification of polyacrylamide gel bands by digital image processing Anal Biochem 1983 133 2 476 81 10.1016/0003-2697(83)90112-4 6638508

[B8] Ye X Suen CY Cheriet M Wang E A Recent Development in Image Analysis of Electrophoresis Gels Vision Interface '99, Trois-Rivières 1999 19-21 432 43823658115

[B9] Machado A Campos M Siqueira M Carvalho O An Iterative Algorithm for Segmenting s in Gel Electrophoresis Images Brazilian Symposium on Computer Graphics and Image Processing - SIBGRAPI 1997 10 140 146

[B10] Griffiths AJF Miller JM Suzuki DT An introduction to genetic analysis 2000 New York: WH Freeman & Co

[B11] Lee J Huang CH Wang NW Lu CS Automatic DNA sequencing for electrophoresis gels using image processing algorithms J Biomed Sci Eng 2011 4 8 523 528 10.4236/jbise.2011.48067

[B12] Gürcan MN Koyutürk M Yildiz HS Cetin-Atalay R Cetin AE Identification of relative protein bands in polyacrylamide gel electrophoresis using a multi-resolution snake algorithm Biotechniques 1999 26 6 1162 6 1168–9 10376155 10.2144/99266bc01

[B13] Rashwan HA Sarhan AM Faheem MT Youssef BA A Novel Approach for Protein Spots Quantification in Two- Dimensional Gel Images International Journal of Signal Processing, Image Processing and Pattern Recognition 2011 4 1 1 15

[B14] Kuick R Boerwinkle E Hanash SM Sing CF A Statistical Analysis of Spot Variation Using the Two-Dimensional Polyacrylamide Gel Electrophoresis Computers And Biomedical Research 1986 19 1 90 102 10.1016/0010-4809(86)90009-1 3948498

[B15] Pizzonia J Electrophoresis gel image processing and analysis using the KODAK 1D software Biotechniques 2001 30 6 1316 20 11414225 10.2144/01306bc06

[B16] Ladner CL Yang J Turner RJ Edwards RA Visible fluorescent detection of proteins in polyacrylamide gels without staining Anal Biochem 2004 326 13 20 10.1016/j.ab.2003.10.047 14769330

[B17] Gel-image extractor software http://www.utoronto.ca/greenblattlab/people/yiliu.htm

[B18] Wang DG Large-scale identification, mapping, and genotyping of single-nucleotide polymorphisms in the human genome Science 1998 280 1077 1082 10.1126/science.280.5366.1077 9582121

[B19] Vohradsky J Panek J Quantitative analysis of gel electrophoretograms by image analysis and least squares modeling Electrophoresis 1993 14 601 612 10.1002/elps.1150140195 8375351

[B20] Umesh PS Flint J An efficient tool for genetic experiments: Agarose gel image analysis Pattern Recognition 2003 36 2453 2461 10.1016/S0031-3203(03)00129-8

[B21] Masra MS Kipli S Zamhari N A Simulation Software for DNA Computing Algorithms Implementation, Muhammad World Academy of Science, Engineering & Technology 2011 72 27123840864

[B22] Xu W Zhai Z Huang K A Novel Universal Primer-Multiplex-PCR Method with Sequencing Gel Electrophoresis Analysis PLoS One 2012 7 1 1 10.1371/journal.pone.0022900 PMC3260127 22272223

[B23] Kim Y Kim JJ Won Y In Y Segmentation of protein spots in 2d gel electrophoresis images with watersheds using hierarchical threshold LNCS - Computer and Information Sciences - ISCIS 2003 2869 389 396

[B24] GelQuant software http://biochemlabsolutions.com/GelQuantNET.html

[B25] Gelanalyzer softwarehttp://www.gelanalyzer.com/

[B26] GelPro software http://www.mediacy.com/index.aspx?page=GelPro

[B27] Decodon softwarehttp://www.decodon.com/

[B28] BioNumerics2D softwarehttp://www.applied-maths.com/bionumerics_2d/bionumerics_2d.htm

[B29] Delta2D softwarehttp://www.gelifesciences.com/

[B30] Melanie softwarehttp://www.genebio.com/products/melanie/

[B31] Bio-Rad softwarehttp://www.bio-rad.com/

[B32] Progenesis Samespots softwarehttp://www.nonlinear.com/products/progenesis/samespots/overview/

[B33] RedFin softwarehttp://www.ludesi.com/redfin/

[B34] Sugahara Y Hayashizaki Y Tanihata I An automatic image analysis system for RLGS_LMS Mammalian Genome 1998 9 643 651 10.1007/s003359900837 9680385

[B35] Takahashi K Nakazawa M Watanabe Y DNA insight: An image processing system for 2-d gel electrophoresis of genomic DNA Genome Informatics 1997 8 135 146 11072313

[B36] Morris JS Clark BN Gutstein HB Pinnacle: a fast, automatic and accurate method for detecting and quantifying protein spots in 2-dimensional gel electrophoresis data Bioinformatics 2008 24 4 529 536 10.1093/bioinformatics/btm590 18194961 PMC2662725

[B37] Korzynska A Iwanowski M Multistage morphological segmentation of bright-field and fluorescent microscopy images Opto-Electronics Review 2012 20 2 174 186 10.2478/s11772-012-0026-x

[B38] Koprowski R Wróbel Z Layers recognition in tomographic eye image based on random contour analysis. Computer Recognition Systems 3, Advances in Intelligent and Soft Computing 2009 57 471 478 10.1007/978-3-540-93905-4_56

[B39] Koprowski R Wrobel Z Zieleznik W Automatic Ultrasound Image Analysis in Hashimoto's Disease Advances In Pattern Recognition Book Series: Lecture Notes in Computer Science 2010 6256 98 106 10.1007/978-3-642-15992-3_11

[B40] Koprowski R Wrobel Z The cell structures segmentation Computer Recognition Systems, Proceedings Book Series: Advances In Soft Computing 2005 Springer Berlin Heidelberg 569 576

[B41] Porwik P Para T Some handwritten signature parameters in biometric recognition process Proceedings of the ITI 2007 29th International Conference on Information Technology Interfaces Book Series: ITI 2007 Cavtat: CROATIA 185 190

[B42] Ijaz UZ Chaudhary SU Don MS Kim KY Computational Strategies for Protein Quantitation in 2D Electrophoresis Gel Image Processor for Matlab Proceedings of the 2007 Frontiers in the Convergence of Bioscience and Information Technologies, FBIT 2007 2007 Jeju Island, Korea 129 134

[B43] Hoeflich CS Corso JJ Segmentation of 2D Gel Electrophoresis Spots Using a Markov Random Field Proceedings of SPIE Conference on Medical Imaging 2009 7259 4O

[B44] Iakovidis DK Maroulis D Zacharia E Kossida S A Genetic Approach to Spot Detection in two-Dimensional Gel Electrophoresis images 2006 Greece: Proceedings of International Conference on Information, Technology in Biomedicine

[B45] Manolakos ES TP Active Contours Based Segmentation of 2DGE Proteomics Images 2008 Lausanne, Switzerland: Proceedings of Eusipco

[B46] Yoon JW Godsill SJ Kang C Kim TS Bayesian Inference for 2D Gel Electrophoresis Image Analysis 2007 Development: Bioinformatics Research and 343 356

[B47] Rashwan S Faheem T Sarhan A Youssef BA A relational fuzzy c-means algorithm for detecting protein spots in two-dimensional gel images 2010 Advances in Computational Biology: Springer 10.1007/978-1-4419-5913-3_25 20865504

[B48] Berth M Moser F Kolbe M Bernhardt J The state of the art in the analysis of two-dimensional gel electrophoresis images Appl Microbiol Biotechnol 2007 76 1223 1243 10.1007/s00253-007-1128-0 17713763 PMC2279157

[B49] Zhou G Li H DeCamp D 2D Differential In-gel Electrophoresis for the Identification of Esophageal Scans Cell Cancer-specific Protein Markers Molecular & Cellular Proteomics 2002 1 117 123 10.1074/mcp.M100015-MCP200 12096129

[B50] Kim G Kim MH 2D Electrophoresis Gel Image and Diagnosis of a Disease CoRR The Computing Research Repository 2003 305048 1 10

[B51] Steven P Aebersold G Aebersold R Absolute Quantitation of 2-D Protein Spots Methods Mol Biol 2008 112 417 421 10.1385/1-59259-584-7:417 10027266

[B52] Otsu N A threshold selection method from gray-level histograms Transactions on Systems, Man, and Cybernetics 1979 9 1 62 66

[B53] Opara K Koprowski R Image segmentation polyacrylamide gels in the analysis of DNA polymorphisms 2006 Poland: Uniwersytet Śląski

[B54] Sonka M Michael Fitzpatrick J Medical Image Processing and Analysis 2000 Belligham SPIE: In Handbook of Medical Imaging

[B55] Caridade CMR Marcal ARS Mendonca T Pessoa AM Pereira S Automatic information extraction from Gel Electrophoresis Images using GEIAS Springer Lecture Notes in Computer Science 2010 6112 185 194 10.1007/978-3-642-13775-4_19

